# Influence of intra‐articular injectate volume on joint surface coverage and leakage following canine elbow arthroscopy: A cadaveric study

**DOI:** 10.1111/vsu.70141

**Published:** 2026-08-06

**Authors:** Erin Smith, Sarah Stark, Shelby Boynton, Wanda Gordon‐Evans

**Affiliations:** ^1^ University of Minnesota Department of Veterinary Clinical Sciences St. Paul Minnesota USA

## Abstract

**Objective:**

To determine whether reduced intra‐articular (IA) injection volumes provide complete synovial surface coverage while minimizing leakage following canine elbow arthroscopy.

**Study design:**

Randomized cadaveric study.

**Animals:**

Forty elbow joints from 20 canine cadavers weighing >15 kg.

**Methods:**

Standard medial elbow arthroscopy was performed with portals placed under arthroscopic guidance. The egress portal was created along the caudal margin of the medial epicondylar ridge, directed toward the anconeal process. A third, craniomedial instrument portal was created approximately 1 cm cranial to the camera portal. Following arthroscopy, India ink—volume‐matched to whole, half, or quarter of a 0.5 mg/kg ropivacaine dose (0.75% concentration)—was injected via the egress portal. Each joint underwent 10 passive flexion–extension cycles before dissection. A masked investigator (ES) qualitatively assessed synovial surface coverage (100%, 75%–99%, 50%–74%, 25%–49%, 0%–24%) and leakage severity (mild, moderate, severe). Portal placement attempts, cadaver age, and bodyweight were recorded.

**Results:**

Mean cadaver age was 9.3 years (range: 1–16 years), and mean weight was 29.9 kg (range: 17.9–43.6 kg). Complete (100%) synovial surface coverage was observed in all 40 elbows, including those receiving quarter‐dose injections. Leakage occurred in every dose group, with a similar distribution of mild, moderate, and severe categories. Portal placement was successful on the first attempt in 32 elbows (80%) and the second attempt in eight elbows (20%).

**Conclusion:**

Reduced IA injection volumes achieved complete synovial surface coverage but did not reduce leakage severity.

**Clinical significance:**

Reduced intra‐articular injection volumes were sufficient to achieve complete synovial surface coverage in this model, suggesting potential flexibility in clinical dosing strategies. Nonetheless, extravasation occurred at all tested volumes, reinforcing that some leakage should be expected in practice.

## 1 INTRODUCTION

Intra‐articular (IA) administration of local anesthetics is a practical technique employed in veterinary orthopedic surgery to provide immediate postoperative analgesia. In veterinary patients, IA injections are particularly useful following elbow and stifle arthroscopy, where postoperative analgesic requirements are substantial. Although peripheral nerve blocks may provide effective perioperative analgesia, their use can require specialized regional anesthesia equipment, such as ultrasound or nerve stimulation, as well as block‐specific technical expertise. In contrast, IA administration can be performed rapidly through an existing arthroscopy portal, making it a practical adjunctive analgesic technique.^1^, ^2^, ^3^, ^4^, ^5^, ^6^, ^7^

Despite its routine use in human medicine, IA injection of local anesthetics remains controversial. A central concern is the risk of chondrotoxicity—particularly with amide‐type local anesthetics such as bupivacaine and lidocaine—which has been shown to induce chondrocyte apoptosis and necrosis in vitro in a dose and time‐dependent manner.^8^, ^9^, ^10^ Jayaram et al. conducted a systematic review evaluating the effects of local anesthetics on human cartilage and concluded that commonly used agents exhibit significant chondrotoxicity, with potential implications for post‐arthroscopic cartilage health.^10^ In veterinary studies, intra‐articular bupivacaine has been shown to provide effective pain relief in dogs undergoing stifle and elbow surgery,^5^, ^6^ but concerns about its cartilage safety have prompted interest in less toxic alternatives.

Ropivacaine, a long‐acting amide local anesthetic with reduced lipid solubility, has emerged as a potentially safer alternative to bupivacaine because multiple experimental studies have demonstrated comparatively reduced chondrocyte toxicity and less activation of apoptotic pathways.^11^, ^12^, ^13^, ^14^, ^15^ Nevertheless, ropivacaine may still exhibit dose‐dependent cytotoxicity, raising the question of how much anesthetic is required to achieve therapeutic benefit while minimizing unnecessary joint exposure.

Beyond cytotoxicity, leakage of injectate from the joint space, especially after arthroscopy, has emerged as a major clinical and scientific concern. Intra‐articular injections administered through recent arthroscopic portals may be prone to extra‐articular leakage due to capsular perforations, soft‐tissue disruption, or the absence of inherent resistance to backflow. Leakage reduces the amount of drug available within the joint space for synovial uptake and local receptor binding, which may compromise the intended analgesic effect and expose periarticular tissues (nerves, vessels, muscle) to unintended drug concentrations.^16^

Leakage has been investigated in two cadaveric studies that showed contrast extravasation in canine elbows is worse after arthroscopy.^16^, ^17^ This is likely due to disruption of capsular and soft tissue barriers. Leakage appears volume‐dependent, yet even 2‐mL injections produced measurable extravasation, suggesting that elbow joint capacity is limited and easily exceeded.^16^ These prior works used fixed volumes rather than weight‐based dosing, did not evaluate joint surface coverage, and did not attempt to identify a minimum effective dose.^16^

The ideal volume and dose required to balance effective surface coverage while minimizing extra‐articular leakage remains unclear. Adequate coverage of the synovial surface is necessary to ensure therapeutic efficacy, but over‐titration of volume may negate this benefit by increasing chondrotoxicity and leakage. Currently, there is no consensus on what constitutes an optimal IA dose of ropivacaine in dogs, nor is it clear how volume reduction may affect either surface coverage or leakage severity in the context of a post‐arthroscopic joint. Given the small size of the canine elbow and its relatively constrained capsule, it is plausible that lower, weight‐based doses may suffice to coat the joint and deliver analgesia while minimizing off‐target effects.

The objectives of the present study were two‐fold: (1) To determine whether reduced injection volumes, based on fractions of a 0.5 mg/kg dose of 0.75% ropivacaine, were sufficient to achieve complete intra‐articular surface coverage following elbow arthroscopy; and (2) to characterize the extra‐articular distribution of injectate following elbow arthroscopy using a controlled cadaveric model. The hypothesis was that (1) reduced injection volumes would still achieve complete synovial surface coverage; and (2) increasing injection volume would be associated with greater extra‐articular injectate migration and involvement of more distant periarticular tissues.

## 2 MATERIALS AND METHODS

### 2.1 Study design and sample population

A randomized, controlled cadaveric study was conducted using 40 elbow joints harvested from 20 adult canine cadavers, each weighing over 15 kg. The sample size was selected based on precedent from Blumhagen et al., who used 40 elbows from 20 canine cadavers to evaluate post‐arthroscopy intra‐articular injectate dynamics.^16^ However, a formal a priori power analysis was not performed for the present study because the primary outcome was gross categorical assessment of synovial surface coverage, and no prior studies using this methodology were available to provide estimates of expected variance or effect size. All cadavers were clinical patients donated post‐euthanasia for educational and research purposes, with cause of death unrelated to the musculoskeletal system. Inclusion criteria required structurally intact elbow joints without prior trauma, surgery, or neoplasia. Cadavers were excluded if lateral condylar fractures, elbow luxations, or periarticular tumors were identified during screening. All cadavers were stored frozen and thawed at room temperature prior to use. Study design and reporting followed standard practices for cadaveric orthopedic research.^16^, ^17^, ^18^, ^19^

### 2.2 Arthroscopic procedure

Each elbow underwent a standard medial elbow arthroscopy as described by Tobias and Johnston and consistent with prior canine cadaveric studies.^18^, ^19^, ^20^ The skin overlying the joint was clipped. A 3‐cc syringe and 22‐gauge needle were inserted just distal and slightly caudal to the medial epicondyle. Proper intra‐articular placement was confirmed by aspiration of synovial fluid. With the needle still in place, lactated Ringer's solution was injected until the joint was visibly distended and back pressure was felt on the syringe plunger, confirming capsular tension. This needle location marked the site for the medial camera portal.

A #11 scalpel blade was used to make a proximo‐distal stab incision at the needle site, followed by insertion of a blunt obturator and arthroscope cannula into the joint capsule. Once it felt in the joint, the obturator was removed, free fluid outflow was observed, and a 2.4 mm 30° arthroscope (Karl Storz Veterinary Endoscopy, Goleta, California) was inserted. A pressurized fluid bag set at approximately 300 mmHg was used to maintain joint distension throughout the procedure.^19^, ^20^

An egress portal was established under direct arthroscopic visualization by inserting an 18‐ or 20‐gauge needle along the caudal margin of the medial epicondylar ridge toward the anconeal process. Entry into the joint was confirmed by a clearing of the arthroscopic view and active fluid flow. A third, craniomedial instrument portal was created approximately 1 cm cranial to the camera portal using the same technique, except the scalpel blade was deliberately advanced into the joint space. This portal was used for diagnostic palpation. (Figure 1).

**FIGURE 1:**
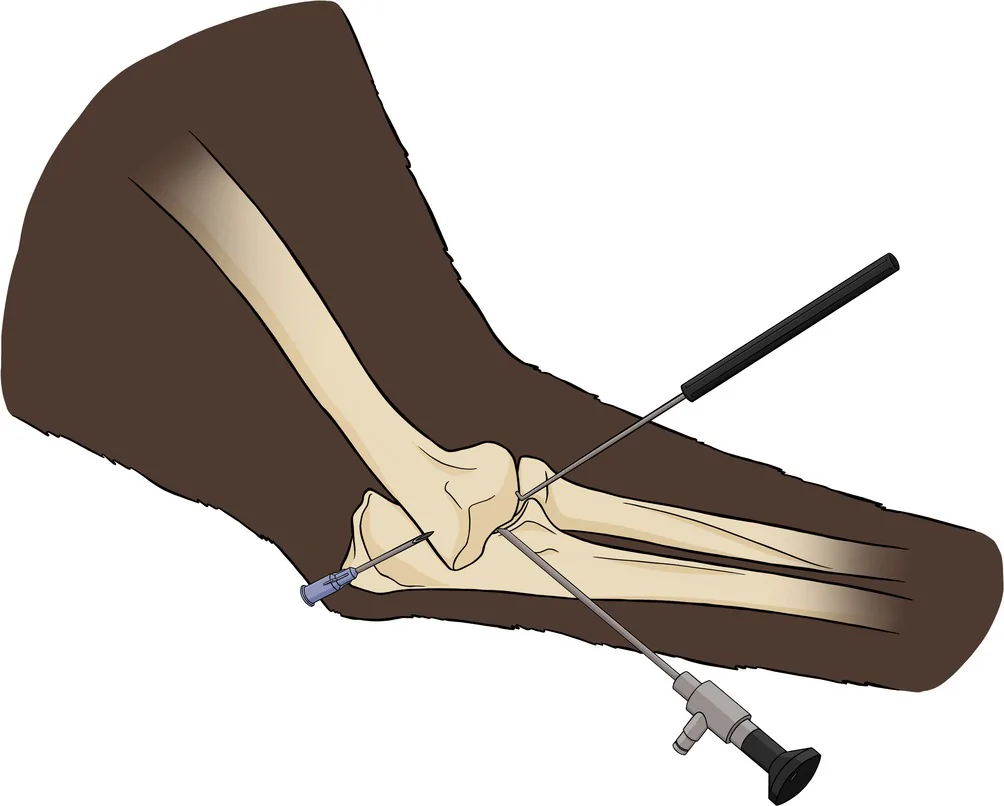
Overview of arthroscopic methodology. Medial view of the canine elbow demonstrating arthroscopy portal placement. The camera portal is positioned just distal and slightly caudal to the medial epicondyle. The egress portal is created along the caudal margin of the medial epicondylar ridge and directed toward the anconeal process. The instrument portal is placed approximately 1 cm cranial to the camera portal.

Each joint was visually inspected and probed using standard arthroscopic instrumentation. No therapeutic procedures such as coronoidectomy or fragment removal were performed. Any difficulty in joint distension, portal placement, visualization, or manipulation was recorded. After completion of the diagnostic arthroscopy, all residual fluid was aspirated from the joint via the egress needle, which remained in place for subsequent injection. All other portals were closed using single cruciate sutures of 3–0 nylon for both subcutaneous and skin layers, following standard closure practices.^18^, ^19^, ^20^

### 2.3 Experimental protocol

Randomization of limb assignment (left or right) was done by random number generator. Even numbers generated were assigned as right, and odd as left. Each dog was randomized to one of two paired dose allocations: whole dose in one elbow and half dose in the contralateral elbow, or quarter dose in one elbow and half dose in the contralateral elbow. Randomization was stratified in blocks of two dogs to ensure equal distribution among treatment groups. This was done because paired elbows are not independent of one another. The investigator performing the procedures was masked (ES) to the dose assignments throughout the study to minimize bias. Whole dose was defined as a volume equivalent to 0.5 mg/kg of ropivacaine (0.75% concentration), and half and quarter dose calculated accordingly. Joint injection was performed via the caudal egress portal that was established under direct arthroscopic visualization. Each joint was then passively flexed and extended 10 times to distribute the solution throughout the joint prior to dissection.

India ink was chosen as the injectate analog because its physical properties approximate those of local anesthetic solutions, allowing it to disperse in a comparable manner within the synovial environment. Its aqueous solubility and low viscosity facilitate diffusion across joint surfaces and through capsular defects, similar to clinical injectates.^21^, ^22^ In addition, its intense pigmentation provides reliable visualization of intra‐ and periarticular spread during dissection. India ink has been used previously in cadaveric models to trace local anesthetic distribution, supporting its validity as a surrogate marker.^23^, ^24^, ^25^

After passive range‐of‐motion cycles were completed, joints were dissected immediately to evaluate injectate distribution. A standard medial approach to the elbow was performed in layers from superficial to deep to allow subsequent arthrotomy and assessment of intra‐articular coverage. During the approach, the skin, subcutaneous tissue, muscle, capsule, and periarticular tissues were sequentially inspected for India ink to determine the most superficial tissue plane reached by the injectate. This tissue plane was used as a surrogate for the distance of extra‐articular ink spread. Extra‐articular ink spread was categorized as mild when India ink was limited to periarticular tissues, moderate when it extended into muscle, or severe when it extended to subcutaneous tissue or skin (Figure 2). By dissecting from superficial to deep, the most superficial layer containing India ink could be identified before iatrogenic disruption of deeper tissues or entry into the joint. Findings were recorded immediately at the time of dissection by a masked investigator to minimize interpretation bias. All dissections and leakage assessments were performed by the same investigator using a standardized technique to maintain consistency among specimens.

**FIGURE 2A & B:**
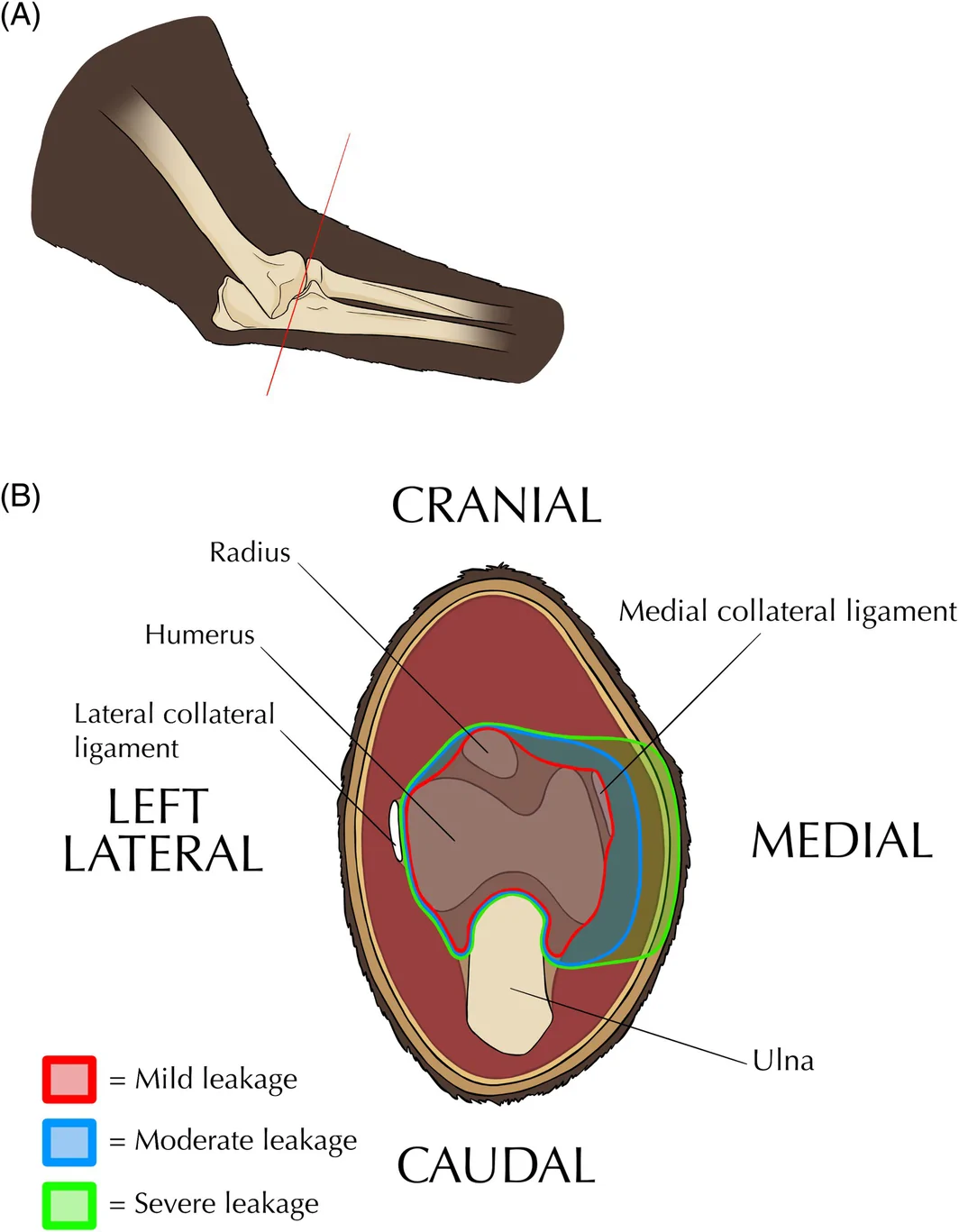
Diagram of maximal extravasation extent for mild, moderate, and severe leakage scores. (A) Medial aspect of the canine elbow illustrating the anatomic location and orientation of the transverse plane used to generate the cross‐sectional view shown in (B). The section was taken through the medial elbow at the level of the joint capsule, perpendicular to the long axis of the limb, to standardize assessment of periarticular fluid spread. (B) Schematic cross‐section of the elbow demonstrating categorical grading of injectate leakage severity based on tissue plane involvement. Minimal leakage (red) was defined as injectate confined to periarticular tissues immediately adjacent to the joint capsule. Moderate leakage (blue) was defined as extension of injectate into surrounding musculature. Severe leakage (green) was defined as spread of injectate beyond muscle planes into the subcutaneous tissues and/or toward the skin surface. Leakage severity was assigned at the time of dissection based on the deepest tissue plane reached by the injectate.

Following dissection to the joint, the elbow was disarticulated by severing the medial collateral ligament and joint capsule, and synovial surface coverage was assessed by gross visual inspection of India ink distribution throughout the joint. Coverage was assigned to one of five predefined ordinal categories representing the estimated proportion of visible synovial surfaces stained by India ink: 0%–24%, 25%–49%, 50%–74%, 75%–99%, or 100%. These categories were based on visual estimation rather than direct surface area measurement or digital image analysis, as the complex three‐dimensional anatomy of the elbow joint, including curved articular surfaces and variable tissue depth, could not be reproducibly converted into a standardized two‐dimensional image for quantitative analysis. All coverage assessments were performed by a single investigator using the same scoring criteria.

### 2.4 Data recording and analysis

Data recorded for each elbow included cadaver age, weight, side (left vs. right), assigned dose group, number of attempts to establish the camera portal, joint surface coverage score, and leakage severity score. Descriptive statistics were generated for all variables. Patterns of coverage and leakage were then reviewed qualitatively to determine whether formal statistical comparisons between dose groups were appropriate.

## 3 RESULTS

A total of 40 elbow joints from 20 canine cadavers were included in the study. All cadavers met inclusion criteria, with no evidence of elbow trauma, neoplasia, or prior orthopedic intervention. The mean age was 9.3 years (range: 1–16 years), and the mean bodyweight was 29.9 kg (range: 17.9–43.6 kg). Mean ± SD injection volumes were 0.50 ± 0.16 mL for the quarter‐dose group, 1.01 ± 0.25 mL for the half‐dose group, and 1.96 ± 0.40 mL for the whole‐dose group. The study population included Labrador Retrievers (*n* = 5), Golden Retrievers (*n* = 2), American Staffordshire Terriers (*n* = 2), Pit Bulls/American Pit Bull Terriers (*n* = 3), and one each of German Shorthair, German Shepherd Dog, English Bulldog, Coonhound, Border Collie, Doberman, Labradoodle, and mixed‐breed Doodle.

Camera portal placement was successfully achieved on the first attempt in 32 of 40 elbows (80%) and on the second attempt in eight of 40 elbows (20%). No elbow required more than two attempts for successful portal establishment. Intra‐articular injection volumes ranged from 0.3 to 2.8 mL. Following intra‐articular injection and passive joint motion, complete (100%) joint surface coverage was achieved in all 40 elbows, consistent across whole, half, and quarter dose groups (Figure 3).

**FIGURE 3:**
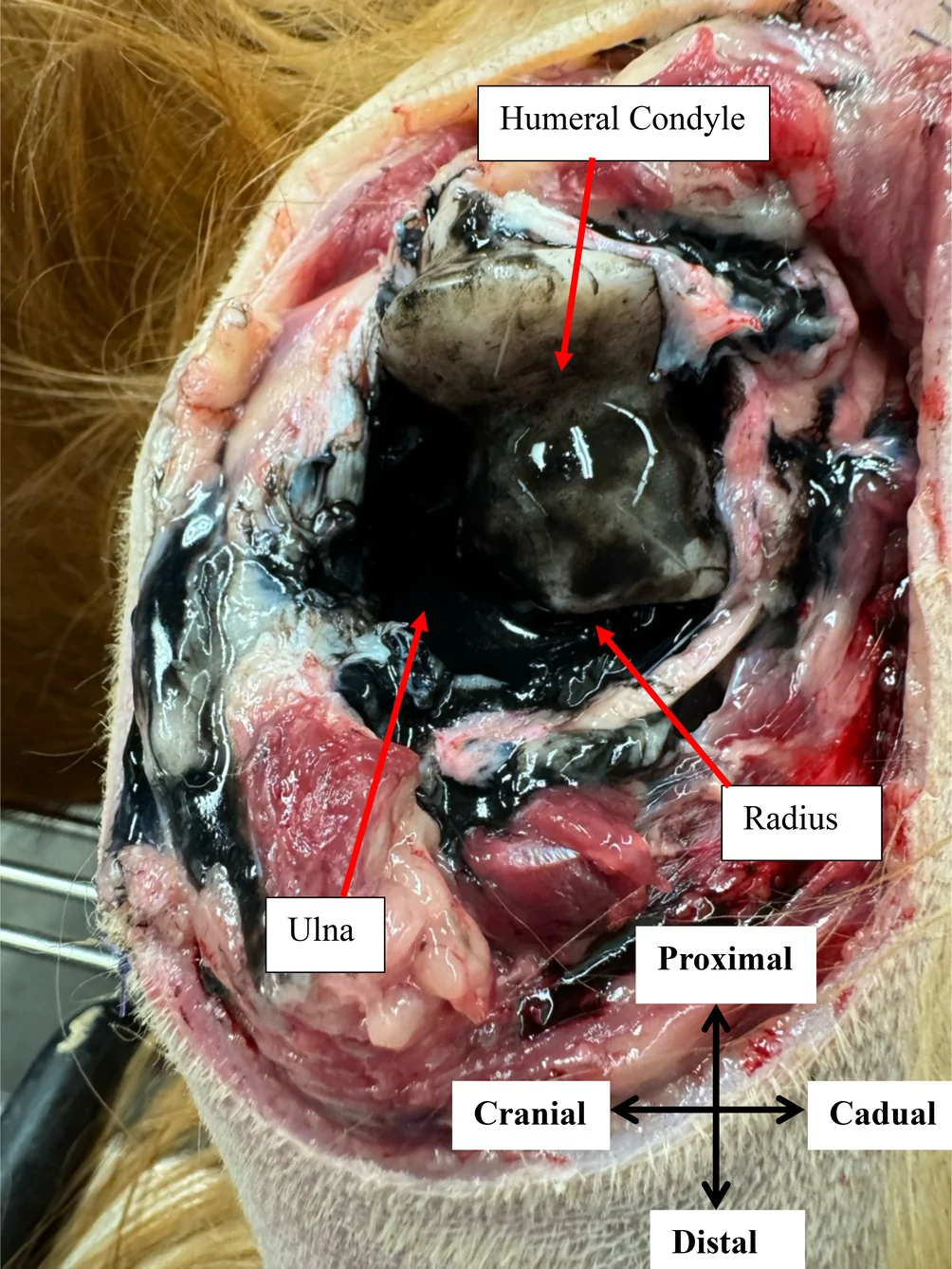
Representative example of complete articular surface coverage. Representative example of complete articular surface coverage. Gross photograph of the canine elbow joint following intra‐articular injection of India ink and subsequent dissection. India ink is visible across all exposed synovial surfaces. Normal articular cartilage does not uptake stain and will have a variable look.^34^ This image represents the highest coverage category used for subjective grading of intra‐articular injectate distribution (100%). The image is oriented with proximal at the top, distal at the bottom, cranial to the left, and caudal to the right.

Leakage of injectate was observed in all dose groups without a discernible pattern (Table 1). The distribution of leakage severity was comparable among the whole, half, and quarter dose groups. In the absence of a dose‐dependent relationship, statistical comparisons were not performed.

**TABLE 1:** Distribution of leakage severity across dose groups following intra‐articular injection after elbow arthroscopy in 20 canine cadavers (40 elbows).

Dose group	Mild leakage	Moderate leakage	Severe leakage	Total elbows
Whole dose	3	4	3	10
Half dose	7	8	4	20
Quarter dose	3	4	3	10

*Note*: Values represent number of elbows in each category. Leakage severity was classified as mild (periarticular only), moderate (extending into muscle), or severe (extending to subcutaneous tissue or skin). Whole dose = 0.5 mg/kg ropivacaine equivalent; half and quarter doses calculated proportionally.

## 4 DISCUSSION

This study investigated whether reduced volumes of intra‐articular (IA) injectate could achieve adequate intra‐articular surface coverage. Additionally, it evaluated the relationship between IA injection volume and the anatomic extent of extra‐articular injectate migration following elbow arthroscopy in a canine cadaver model. The first hypothesis was supported: the quarter‐dose volume matched to India ink consistently achieved complete joint surface coating in all specimens. In contrast, contrary to the second hypothesis, results revealed no apparent association between dose volume and the extent of extra‐articular injectate migration. These findings suggest that IA analgesic protocols could potentially be volume‐reduced without sacrificing intra‐articular distribution.

Complete coverage at the lowest volume evaluated suggests that smaller volumes may warrant investigation; however, additional dose reduction was not evaluated because a 25% fraction of the conventional dose was considered a substantial reduction at the time of study design. Further dose reduction may be possible, but there may be a trade‐off in length or level of efficacy.

Blumhagen et al.^16^ reported a statistically significant increase in leakage scores with 4‐mL injections compared to 2‐mL injections, based on computed tomography (CT)‐derived volumetrics and blinded extravasation scoring. Rustemeyer et al.^17^ similarly demonstrated extensive leakage of contrast following arthroscopy compared with arthrocentesis. In contrast, the present study found no dose‐dependent leakage trend, despite the use of a wide range of bodyweights and volume‐scaled doses calculated from 0.5 mg/kg of 0.75% ropivacaine. Importantly, injection volumes in this study (0.3–2.8 mL across all groups) were smaller than those reported by Blumhagen et al.,^16^ and likely remained within the physiologic capacity of the canine elbow. This difference may explain the absence of a volume–leakage correlation. Variation in assessment techniques, direct gross dissection in the present study versus CT volumetrics in prior reports, may also account for the contrasting outcomes.

Rustemeyer et al.^17^ also found that leakage was immediate and did not progress over a 15‐min period. Although the present study did not assess a time effect, these findings suggest that the full extent of leakage was already present at the time of dissection.

The clinical consequences of leakage are highly substance dependent. For corticosteroids, hyaluronic acid, and biologics, therapeutic benefit depends on sustained intra‐synovial residence. Extravasation in these cases represents not only a therapeutic loss but also potential adverse sequelae, including periarticular tissue irritation, fibrosis, or reduced cost‐effectiveness of expensive biologics.^26^, ^27^ In contrast, periarticular leakage of local anesthetics may confer clinical benefit. When these drugs diffuse into surrounding tissues, they can act on nociceptors within the joint capsule, musculature, and subcutaneous tissues, thereby broadening their analgesic effect across the portal tract.^4^, ^5^, ^6^, ^28^ This combined intra‐ and periarticular mechanism is consistent with multimodal pain management and may be particularly advantageous in cases involving significant capsular manipulation or soft tissue trauma. Clinical studies in dogs undergoing orthopedic surgery have demonstrated improved analgesia when IA local anesthetics are administered, even with likely periarticular spread.^4^, ^5^, ^6^

These contrasting implications underscore the importance of tailoring surgical and injection strategies to the intended therapeutic agent. For local anesthetics, periarticular extravasation may augment analgesia. In contrast, for corticosteroids, hyaluronic acid, and orthobiologics, extravasation undermines efficacy and safety. Therefore, meticulous portal management and joint capsule closure would be logically considered when postoperative injections of corticosteroids or biologics are planned.

One of the most impactful findings of the present study was that even quarter‐dose injections achieved 100% synovial and articular surface coverage. This provides evidence from an ex vivo cadaveric model that meaningful volume reduction may still achieve complete intra‐articular distribution; however, clinical analgesic efficacy cannot be inferred from these findings. Nevertheless, cadaveric models represent an important preliminary step in evaluating intra‐articular drug delivery strategies before clinical efficacy studies are undertaken.^16^, ^17^, ^29^, ^30^ Such findings are clinically relevant given the established dose‐related toxicity of local anesthetics.^8^, ^9^, ^10^, ^11^, ^12^ An additional consideration is that residual arthroscopic lavage fluid may remain within the joint following surgery despite aspiration through the egress needle. This residual fluid may dilute the injected ropivacaine, thereby lowering the effective intra‐articular concentration and potentially influencing both injectate distribution and leakage patterns. In the present study, the arthroscopic procedure and aspiration technique were standardized across specimens, and all elbows were aspirated through the egress needle before India ink injection. Therefore, although residual lavage fluid may have influenced absolute leakage severity or dilution, this effect would be expected to have occurred similarly across treatment groups, allowing relative comparisons among injection volumes to remain interpretable. Such dilution could theoretically reduce chondrotoxic risk by decreasing chondrocyte exposure to higher local anesthetic concentrations; however, it also introduces uncertainty regarding the true concentration of anesthetic delivered to chondrocytes and nociceptors. This is clinically relevant because intra‐articular local anesthetic efficacy and chondrotoxicity appear to be influenced by drug concentration, exposure duration, and joint pharmacokinetics. In human medicine, dry arthroscopy has been proposed as an alternative technique for joint evaluation and treatment.^31^, ^32^ To our knowledge, this technique has not been reported, validated, or standardized in dogs. Future investigations evaluating arthroscopic fluid management, residual lavage volume, and alternative arthroscopic techniques may further improve understanding of factors influencing intra‐articular drug retention and delivery following arthroscopy. The relationship between injected concentration, effective intra‐articular concentration, and clinical analgesic efficacy remains undefined, and a randomized, blinded, controlled clinical trial is warranted to evaluate the in vivo efficacy of a quarter‐dose protocol.

This study has several important limitations, including the cadaveric design, semi‐quantitative outcome measures, and use of India ink as a surrogate for injectate distribution. The cadaveric model precluded assessment of physiologic factors that may influence intra‐articular drug distribution and persistence in clinical patients, including synovial fluid turnover, vascular absorption, inflammatory responses, clotting, and active joint motion.^31^ Freeze–thaw cycles may also alter soft tissue characteristics, capsular integrity, and tissue permeability, potentially affecting injectate distribution and leakage. However, all specimens underwent the same freeze–thaw protocol and were handled in a standardized manner, allowing relative comparisons among treatment groups. Most importantly, although synovial surface coverage was used as a surrogate for potential drug distribution, analgesic efficacy could not be evaluated in this model.

Leakage was assessed by gross dissection using an ordinal grading system rather than by quantitative imaging. Although dissection allowed direct visualization of the tissue planes involved and reflected a clinically relevant method of assessing gross leakage, it did not permit volumetric quantification of extravasated injectate. Advanced imaging modalities, such as CT, may allow more precise measurement of leakage volume and would permit more direct comparison with prior CT‐based studies. However, the objective of the present study was to categorize the anatomic extent of leakage rather than to quantify total extravasated volume; therefore, a structured ordinal scoring system was considered appropriate for this model.

India ink was selected as the injectate surrogate because it has been used previously in cadaveric models to evaluate local anesthetic spread during dissection studies.^23^, ^24^, ^25^ However, India ink does not reproduce the pharmacologic properties, tissue binding, diffusion kinetics, or biologic interactions of ropivacaine with synovium.^33^ Interpretation of India ink distribution across articular cartilage also requires caution. India ink preferentially accumulates within surface irregularities, fibrillation, and microscopic defects, whereas intact cartilage surfaces retain little visible dye.^34^ Therefore, variation in staining intensity across articular cartilage, as seen in Figure 3, should not be interpreted as incomplete injectate distribution. In the present study, coverage assessment was based on the visible presence of India ink throughout the joint and across exposed synovial surfaces, rather than on uniform staining intensity of the cartilage itself. If ropivacaine follows a similar distribution pattern, reduced retention on intact articular cartilage could theoretically limit direct chondrocyte exposure. However, this remains speculative because ropivacaine distribution, tissue binding, and cartilage exposure were not directly measured in the present study.

Digital image analysis could theoretically provide a more objective method of estimating surface coverage; however, its application in this model would be limited by the complex three‐dimensional geometry of the elbow joint, specimen variability, differences in tissue depth, and the difficulty of accurately representing curved dissected surfaces using two‐dimensional photographs. In addition, because complete gross coverage was observed in all specimens, quantitative image analysis would be unlikely to provide meaningful additional discrimination among treatment groups.

The absence of an a priori power analysis is another limitation of this study. Sample size was selected with reference to prior cadaveric work; however, the outcome measures differed between studies, limiting direct application of that power calculation to the present model. Specifically, the present study used gross categorical assessment of synovial and articular surface coverage and leakage extent rather than CT‐derived volumetric leakage measurements. Therefore, although complete coverage was observed in all specimens, the study may have been underpowered to detect subtle differences in leakage patterns among dose groups. Complete coverage at the lowest volume evaluated also suggests that smaller volumes may warrant investigation; however, additional dose reduction was not evaluated because a 25% fraction of the conventional dose was considered a substantial reduction at the time of study design. Finally, complete gross coverage should not be interpreted as evidence that the delivered dose or concentration is sufficient to provide clinical analgesia.

Taken together, these limitations indicate that the present findings should be interpreted as proof‐of‐concept evidence of mechanical feasibility rather than evidence of clinical equivalence. Prospective, blinded, in vivo studies are required to determine whether reduced‐volume intra‐articular ropivacaine provides effective analgesia while maintaining an acceptable safety profile in clinical patients.

In conclusion, this cadaveric study demonstrated that reduced‐volume intra‐articular injections following canine elbow arthroscopy achieved complete synovial surface coverage without increasing leakage severity. These findings provide proof‐of‐concept evidence that reduced intra‐articular injectate volumes can still achieve complete synovial surface coverage in a cadaveric elbow model. This demonstrates the feasibility of volume reduction without compromising intra‐articular distribution. The clinical significance of these results lies in informing future investigation of lower‐volume analgesic protocols; however, validation of efficacy, safety, and chondrotoxicity risk require prospective in vivo studies. For medications where extracapsular leakage is undesirable, methods to mitigate leakage should be instituted.

## AUTHOR CONTRIBUTIONS

Smith E, DVM: Contributed to the design of the study, compiled all data, interpreted data, drafted and revised the manuscript. Stark S, DVM: Oversaw data collection and provided scientific, in‐line editing of the manuscript. Boynton S: Contributed to the conceptualization, design, and creation of original scientific illustrations used in this manuscript. Gordon‐Evans W, DVM, PhD, DACVS (Small Animal), DACVSMR: Contributed to the design of the study, interpreted data, and provided scientific, in‐line editing of the manuscript. All authors provided a critical review of the manuscript and endorse the final version. All authors are aware of their respective contributions and have confidence in the integrity of all contributions.

## FUNDING INFORMATION

This study was supported by the Tata Orthopedic Research Endowment.

## CONFLICT OF INTEREST STATEMENT

The authors declare no conflicts of interest related to this study.

## Data Availability

Data generated during this study are available from the corresponding author upon reasonable request.
